# Benchmark Instability in Fractal Dimension Estimation: Distortion Induced by Gray-Level Mapping in Synthetic FBM Images

**DOI:** 10.3390/e28080858

**Published:** 2026-08-01

**Authors:** Wenxuan Jiang, Ze Wang, Xiaoning Jiang, Ji Wang

**Affiliations:** 1 Dalian Scientific Test and Control Technology Institute, Dalian 116013, China; 2 School of Naval Architecture and Ocean Engineering, Dalian University of Technology, Dalian 116024, China; 3 Collaborative Innovation Centre for Advanced Ship and Deep-Sea Exploration, Shanghai Jiaotong University, Shanghai 200240, China; 4 State Key Laboratory of Structural Analysis for Industrial Equipment, Dalian 116024, China

**Keywords:** fractal dimension, synthetic benchmark, linear gray-level mapping, differential box-counting, fractional Brownian motion, algorithm validation

## Abstract

Synthetic fractional Brownian motion (FBM) images serve as standard data for assessing fractal dimension (FD) estimation techniques. The synthesis pipeline transforms continuous FBM matrices into 8-bit grayscale images using linear mapping and quantization, a process often regarded as benign and rarely documented. If this procedure distorts FD estimations, algorithm comparisons based on such benchmarks merge performance with preprocessing errors. We demonstrate that grayscale conversion induces systematic distortion in FD estimation. Identical matrices were initially processed using three linear mapping strategies with varying emphases (direct, 3*σ* statistical, external-coefficient) and subsequently assessed with four FD algorithms (two DBC variants, Higuchi, PSD). The results demonstrate that linear mapping significantly alters FD estimates. In particular, the FD regression slope of the PSD approach notably decreased from 0.9955 (direct mapping) to 0.6400 (external-coefficient mapping), whereas Higuchi displayed negligible sensitivity. The near-perfect log-log linearity ruled out scaling breakdown. The mapping strategies produce distinct grayscale statistical properties that amplify quantization residuals. We present the relative residual to measure this amplification. The relative residual correlates strongly with FD deviations for DBC and PSD methods (*r* up to 0.97), while showing limited association with the Higuchi estimator. These results violate the assumption of benchmark neutrality in FBM-based FD assessment. FD benchmarking studies should, therefore, report preprocessing strategies and minimize relative residuals to ensure algorithmic comparability.

## 1. Introduction

Image processing techniques have garnered increasing research attention across diverse application domains in recent years [1,2,3,4,5]. Within this context, fractal dimension (FD) provides a quantitative measure used for characterizing structural complexity in grayscale images, capturing irregularity, roughness, and scale-invariant spatial organization [6,7]. Its application spans various fields, including medical diagnosis through texture measurement [8,9,10] and surface characterization in material science [11,12]. The reliability of FD-based results in these areas fundamentally relies on the precision, robustness, and methodological clarity of FD estimation methods.

Several frameworks for FD estimation have been developed for the analysis of grayscale images. The differential box-counting (DBC) method [13,14,15,16] is widely used owing to its intuitive geometric foundation and computational efficiency. The Power Spectral Density (PSD) approach [17,18] calculates fractal dimension (FD) from the decay slope of the Fourier power spectrum, while the Higuchi method [19,20] utilizes local intensity differences in the spatial domain. These methods are designed to produce precise FD estimates, despite their divergent theoretical bases.

To conduct rigorous validation and comparative evaluation of FD estimators, benchmark datasets with analytically known theoretical fractal dimensions (TFDs) are required. In practice, synthetic two-dimensional fractional Brownian motion (FBM) images have emerged as the standard reference [13,21]. Their TFD is deterministically related to the Hurst exponent *H* using the expression *TFD* = 3 − *H*, providing an unambiguous ground-truth criterion for algorithmic assessment [22]. However, generating an FBM benchmark image requires an intermediate step: the continuous FBM matrix must be transformed into an 8-bit grayscale image using linear gray-level mapping and integer quantization. Crucially, most FBM-based validation studies do not explicitly detail the particular mapping parameters employed in this conversion, namely, the scaling factor and offset [13,19,23,24,25]. Furthermore, prior research has consistently demonstrated systematic differences between estimated FD values and their theoretical counterparts [13,26]. The linear mapping strategy is routinely treated as a formatting detail. This underlying assumption presumes that alternate mapping options have either minimal or equally distributed impacts across different FD estimation techniques and so do not affect relative performance rankings.

This assumption, however, has not been systematically examined. Previous research has shown suggestive but incomplete evidence. Soille and Rivest showed that proportionate gray-level scaling resulted in algorithm-dependent changes in the FD estimates [27]. Panigrahy et al. reported that the gray-level offset affected the stability of DBC variants [13]. Collectively, these findings undermine the concept of mapping neutrality. These studies, however, have concentrated on the isolated perturbations of scaling or offset. The combined effect of the complete preprocessing chain, which includes coefficient determination, scaling, offset, and quantization, has not been systematically investigated. Three questions remain unresolved. First, it has not been determined whether the selection of common mapping strategies (differing in how coefficients are obtained) results in quantifiable changes in FD estimations for the same underlying FBM surface. Second, it is unknown how mapping-induced changes in grayscale statistical characteristics result in algorithm-specific estimation errors. Third, it has not been examined whether a single metric can reflect the influence of mapping across algorithms with diverse computational principles.

To address these gaps, this study conducts a systematic evaluation of linear gray-level mapping within the full FBM benchmark generation workflow, including coefficient selection, scaling, offset, and quantization. We demonstrate that the linear mapping step introduces an overlooked source of statistical distortion that violates the commonly assumed neutrality of FBM-based benchmarks.

Specifically, this work makes three contributions: (1) a quantitative assessment of how three representative gray-level mapping strategies affect FD estimation accuracy across four widely used algorithms; (2) empirical evidence that mapping-induced perturbations in grayscale statistics and quantization residuals produce systematic, algorithm-dependent FD bias, even in cases where the log-log regression exhibits near-perfect linearity; and (3) the introduction of a relative residual metric that explicitly links preprocessing-induced statistical distortion to FD estimation error, enabling cross-algorithm comparison and interpretation.

These findings establish gray-level mapping as a confounding variable in FBM-based FD evaluation and underscore the necessity of explicit reporting and control of preprocessing strategies in benchmark-driven fractal analysis.

## 2. Materials and Methods

### 2.1. Synthesis of FBM Images

Two main algorithms are commonly used to synthesize two-dimensional FBM surfaces: spatial-domain recursive displacement methods, such as the midpoint displacement method and its variants, and frequency-domain methods based on Fourier filtering [28,29]. Although midpoint displacement is computationally efficient and intuitive, it has a fundamental limitation: when the Hurst exponent H≠1/2, it does not generate a true FBM field with stationary increments [28]. This nonstationarity may cause the measured fractal dimension of the generated surface to deviate systematically from the theoretical value D=3−H, making the method unsuitable for benchmark images requiring controlled fractal dimensions.

By comparison, the Fourier filtering method is directly derived from the spectral definition of FBM. It can reproduce the target fractal behavior while avoiding the grid artifacts and nonstationary effects often introduced by recursive displacement schemes [28,30]. Therefore, Fourier filtering was selected as the image synthesis method in this study.

Two-dimensional FBM surfaces were generated using the Fourier filtering method based on the procedure described by Saupe [28]. In the frequency domain (u,v), the power spectrum follows $S(u,v)\propto 1/(u^{2}+v^{2}{)}^{H+1}$. For a two-dimensional FBM surface, the fractal dimension *D* and the Hurst exponent *H* satisfy *D* = 3 − *H*. Thus, the Hurst exponent can be calculated from a given fractal dimension. To generate an image of a specified size and reduce periodic boundary effects, a larger matrix was first synthesized, and the central region was then cropped as the final FBM matrix ***X*** [28].

A fixed parameter set was used to generate the FBM image sequence: theoretical fractal dimension (TFD) values ranging from 2.1 to 2.9 (in steps of 0.1), with the corresponding Hurst exponent *H* decreasing from 0.9 to 0.1 accordingly, and the image size fixed at 512 × 512 pixels. This parameter setting provides a controlled benchmark dataset for evaluating the influence of linear mapping preprocessing on FD estimation.

### 2.2. Linear Mapping Preprocessing Schemes

The original FBM matrix X was a floating-point matrix and must be converted into an 8-bit unsigned grayscale image Z before image-based FD estimation. This conversion was defined as Z = uint8(*a*X + *b*), where *a* and *b* are the linear mapping coefficients. The uint8() is a MATLAB (R2024a) function that converts a matrix into 8-bit grayscale values. The mapped values were constrained to the valid 8-bit range [0, 255] solely by adjusting the linear mapping coefficients, without any extreme value clipping. Three mapping strategies with distinct emphases were introduced in this study.

#### 2.2.1. Direct Mapping

Direct mapping maximizes the available grayscale dynamic range of each image. The source range was defined by the minimum ($X_{min}$) and maximum ($X_{max}$) values of the original matrix X, and the target dynamic range was fixed as 0–255 [31]. The mapping coefficients are as follows:

(1) $$a=\frac{255}{X_{max}-X_{min}},\;b=-a\cdot X_{min}$$

This strategy ensures the highest native contrast for each generated image.

#### 2.2.2. 3σ Statistical Mapping

To obtain a stable and controlled grayscale distribution and reduce the influence of extreme values on image contrast, a 3*σ* statistical mapping strategy was utilized. Assuming that the matrix values approximately follow a Gaussian-like distribution, the source interval was defined as $\left[\mu_{X}-3\sigma_{X},\mu_{X}+3\sigma_{X}\right]$, where $\mu_{X}$ and $\sigma_{X}$ were the mean and standard deviation of X. This interval was mapped to a target range [$L_{low},L_{high}]$, which was narrower than the full 8-bit interval. The unused headroom at both ends of the 8-bit range accommodated the extreme values falling outside the ±3*σ* source interval without clipping. The mapping coefficients are as follows:

(2) $$a=\frac{L_{high}-L_{low}}{\left(\mu_{X}+3\sigma_{X})-(\mu_{X}-3\sigma_{X}\right)},\;b=L_{low}-a\cdot (\mu_{X}-3\sigma_{X})$$

#### 2.2.3. External-Coefficient Mapping

The external-coefficient strategy enforces consistent grayscale mapping coefficients across all images in the same batch. It is designed to preserve the relative amplitude scale and statistical comparability among FBM matrices with different TFDs.

First, the matrix with the largest dynamic range in the batch was identified. Its mapping coefficients were obtained using the direct mapping strategy, so that its values spanned the full [0, 255] range. The same coefficients, denoted by $a^{*}$ and $b^{*}$, were then applied to all the other matrices in the batch:

(3) $$Z=uint8(a^{*}X+b^{*})$$

This strategy ensures that the mapped images preserve the statistical differences among the original FBM images in this study. The trade-off is that matrices with smaller dynamic ranges may not fully span the 8-bit grayscale range.

### 2.3. Fractal Dimension Estimation Algorithms

To evaluate the robustness and sensitivity of FBM surface FD estimation under different linear mapping strategies, four representative algorithms were selected. These methods encompass different theoretical principles and computational mechanisms: Su2025-DBC and Panigrahy2020-DBC represent two improved differential box-counting (DBC) approaches; the two-dimensional Higuchi method is based on difference-based scaling and is considered relatively robust to linear gray-level transformations; the PSD method estimates FD from frequency-domain power-law behavior.

#### 2.3.1. Su2025-DBC Method

The Su2025-DBC method is an improved differential box-counting algorithm proposed by Su et al. [15]. It incorporates the weighting strategy introduced by Jiang et al. [22] to handle incomplete grids and more fully utilize local and global grayscale information.

For an N×N grayscale image, a sequence of consecutive integer grid sizes s was selected within a predefined interval $s_{min}\;\leq \;s\;\leq {\;s}_{max}$. The scale ratio was defined as r=s/N. For each grid (m,n), the number of boxes required to cover the local grayscale variation was calculated from the local extrema $g_{max}$ and $g_{min}$, together with the global grayscale bounds $G_{max}$ and $G_{min}$. The equations for h and $n_{r}$ calculation are as follows:

(4) $$h=\frac{s\times (G_{max}-G_{min}+1)}{N}$$

(5) $$n_{r}(m,n)=W\times \left(\left\lceil \frac{g_{max}}{h}\right\rceil -\left\lceil \frac{g_{min}}{h}\right\rceil +1\right)$$

In Equation (5), a weighting factor W is introduced to account for incomplete boundary grids:

(6) $$W=\frac{A(m,n)}{s\times s}$$

where A(m,n) denotes the actual pixel area covered by the grid. The total number of boxes $N_{r}$ at scale s was obtained by summing over all grids. The fractal dimension was then estimated from the slope of the linear regression between log(1/r) and $log(N_{r})$.

#### 2.3.2. Panigrahy2020-DBC Method

The method proposed by Panigrahy et al. [16] is another improved DBC method. Its key refinements include a GDI (Grid size is a divisor of image size) grid-size selection strategy, an xy-plane shifting mechanism, an adaptive box height based on image eigenvalues, and weighted least-squares regression.

For a given grayscale image, grid sizes s were selected from scales that exactly divide the image size N. To mitigate box-undercounting problem, each grid was evaluated at its original position and after three types of one-pixel shift—to the east, south, and southeast. The maximum box count among these four positions was taken as the grid’s contribution. The box height *h* was determined from the eigenvalue characteristics of the grayscale matrix. The formula for computing $n_{r}$ is as follows:

(7) $$n_{r}(m,n)=\left\lceil \frac{g_{max}-g_{min}+1}{h}\right\rceil$$

In Equation (7), *g_max_* represents the maximum grayscale value within the grid, and *g_min_* represents the minimum grayscale value within the grid.

Finally, the log-log data were fitted using weighted least squares, where the weights were assigned by a trapezoidal membership function to emphasize more reliable intermediate scales.

#### 2.3.3. Two-Dimensional Higuchi Method

Ahammer et al. [19] extended the classical one-dimensional Higuchi algorithm to two-dimensional images by estimating FD from grayscale difference areas at different spatial delays. For an N×N image, a delay parameter k was specified. For each starting index pair (n,m), the local absolute grayscale differences among neighboring pixels ($x_{i,j}^{k},x_{i-1,j}^{k},x_{i,j-1}^{k},x_{i-1,j-1}^{k}$) separated by k were computed to obtain the difference area $A_{n,m}(k)$. The areas over all $k^{2}$ starting positions were then averaged to obtain A(k). The fractal dimension was estimated from the log-log relationship between A(k) and k.

#### 2.3.4. Power Spectral Density Method

The PSD method estimates FD from the power-law decay of a self-affine surface in the spatial-frequency domain. First, the two-dimensional power spectral density of the grayscale matrix was computed as:

(8) $${PSD}_{2D}(f_{x},f_{y})=\frac{1}{L^{2}}{\left|\sum_{m=1}^{N}\sum_{n=1}^{N}Z_{mn}e^{-2\pi i\Delta L(f_{x}m+f_{y}n)}(\Delta L{)}^{2}\right|}^{2}$$

where *L*^2^ is the surface area, and *N* is the number of data points per line/row. *Z_mn_* is the surface profile height at the position (*m*,*n*). ΔL is (*L*/*N*). *f_x_* and *f_y_* are the spatial frequencies in the *x* and *y* directions. Radial averaging was then used to convert the two-dimensional spectrum into a one-dimensional relationship between radial spatial frequency f and average spectral power PSD(f) [17,32]. The resulting spectrum was fitted using the k-correlation (ABC) model [17] over the frequency range:

(9) $${PSD}_{ABC}(f)=\frac{A}{{\left(1+B^{2}f^{2}\right)}^{\frac{C+1}{2}}}$$

In this model, *A* controls low-frequency amplitude, *B* is associated with the characteristic transition frequency, and *C* describes the high-frequency decay slope. For a two-dimensional self-affine surface, the fractal dimension was obtained from the fitted slope parameter D=(7−C)/2(1≤C≤3). This method is particularly suitable for surfaces with clear self-affine spectral behavior.

### 2.4. Evaluation Metrics

#### 2.4.1. Metrics for Assessing FD Estimation Accuracy

Referencing previous studies [13,22], the evaluation of fractal dimension involves two aspects: the comparison of calculated FD values with TFD and the fitting performance during FD estimation in the log-log plot.

First, FD systematic bias was evaluated by regressing the estimated fractal dimension $D_{est}$ against TFD for each algorithm-preprocessing combination. An ideal estimator should satisfy $D_{est}=TFD$, with a slope of 1 and an intercept of 0. The correlation coefficient r, regression slope, and intercept were extracted to evaluate trend consistency, response sensitivity, and systematic offset, respectively.

Second, FD deviations from the theoretical value were quantified using signed error (SE). Let $D_{est}$ denote the estimated FD and TFD the theoretical FD. The signed error is defined as $SE=D_{est}-TFD$.

Third, the intrinsic scaling reliability of each algorithm was evaluated using the goodness-of-fit $R^{2}$ from its internal log-log regression. A higher $R^{2}$ indicates that the scaling data used for FD estimation adhere more closely to a linear relationship in log-log fitting. Comparing $R^{2}$ across preprocessing strategies helps assess whether linear mapping disrupts the scale-dependent structure required by each algorithm.

#### 2.4.2. Metrics for Assessing Linear Mapping Fidelity

Global statistical changes were assessed by comparing the mean and standard deviation of the original matrix X and the mapped image Z. These comparisons quantified how different linear mapping strategies altered the statistical moments of the image and supported the subsequent analysis of FD estimation behavior.

The Pearson correlation coefficient between the original matrix X and the final grayscale image Z was used to assess structural consistency. A value close to 1 indicates that the relative surface fluctuations in the original FBM matrix are well preserved after mapping.

Residual statistics were used to quantify numerical errors introduced by linear mapping and integer quantization. The residual was defined as the difference between the theoretical mapped value Y (=*a*X + *b*) and the final grayscale value Z. The residual mean measures systematic bias, whereas the residual standard deviation reflects random quantization error or nonlinear distortion. Ideally, the mean and standard deviation of the residuals should be zero.

### 2.5. Overall Experimental Workflow

**Figure 1:**
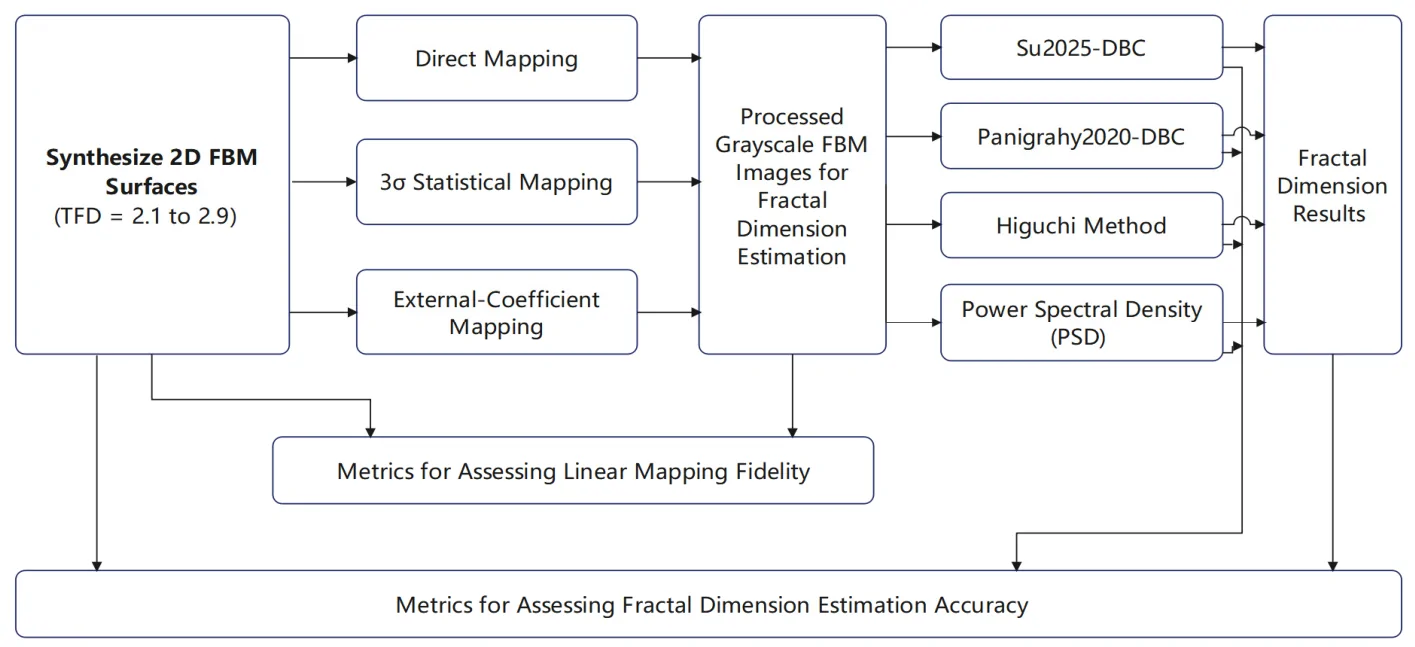
Experimental workflow.

Figure 1 summarizes the experimental workflow used to evaluate the influences of linear mapping strategies across FD estimation algorithms.

**Figure 2:**
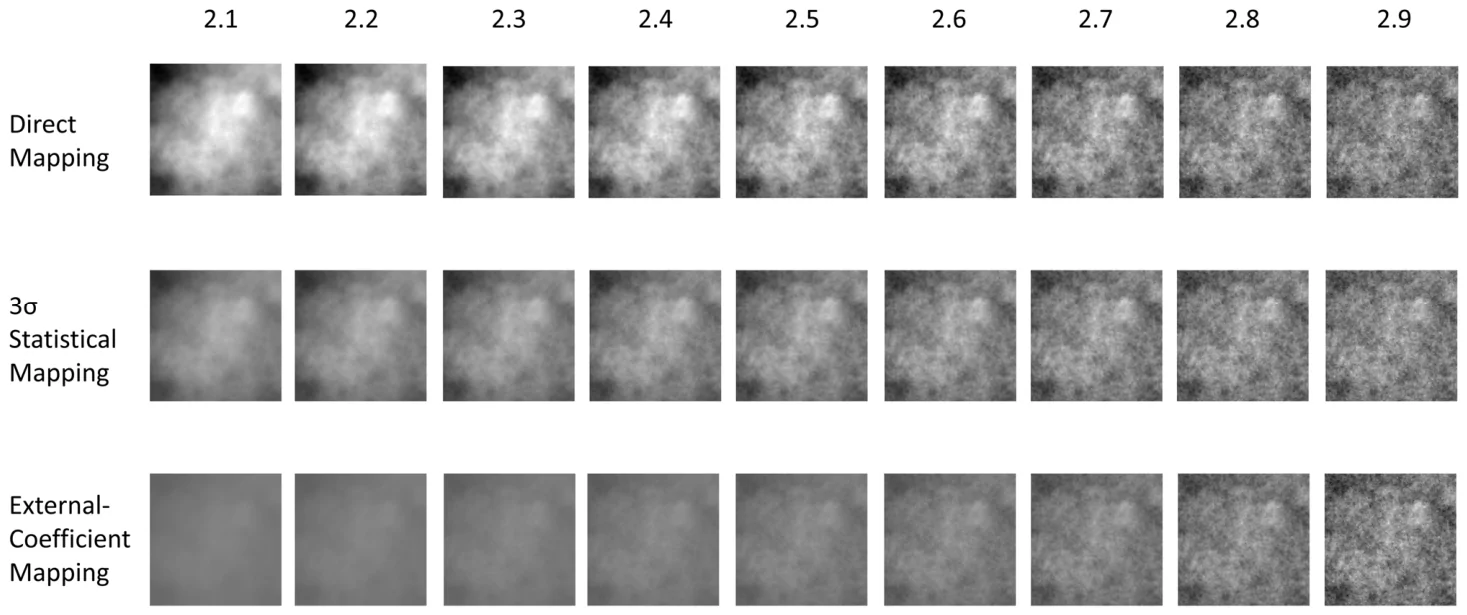
Processed FBM images for fractal dimension estimation.

The workflow consists of three stages. First, two-dimensional FBM surfaces with known theoretical fractal dimensions ranging from 2.1 to 2.9 were generated. The raw FBM matrix was a floating-point array and could not be directly displayed as a grayscale image. To convert it into a visible grayscale image, each original matrix was processed using three linear mapping strategies: direct mapping, 3*σ* statistical mapping, and external-coefficient mapping. The FBM grayscale images preprocessed by the three linear mapping strategies are shown in Figure 2. Second, all preprocessed images were analyzed using the four FD estimation algorithms. Third, evaluation was performed at two levels: FD estimation performance and the fidelity of the linear mapping.

## 3. Results

### 3.1. FD Estimation Results and Error Analysis

To evaluate the influence of linear mapping preprocessing on fractal dimension estimation, FBM grayscale images generated by the three mapping strategies were analyzed using four FD estimation algorithms. The estimated fractal dimensions were then compared with the theoretical values.

**Figure 3:**
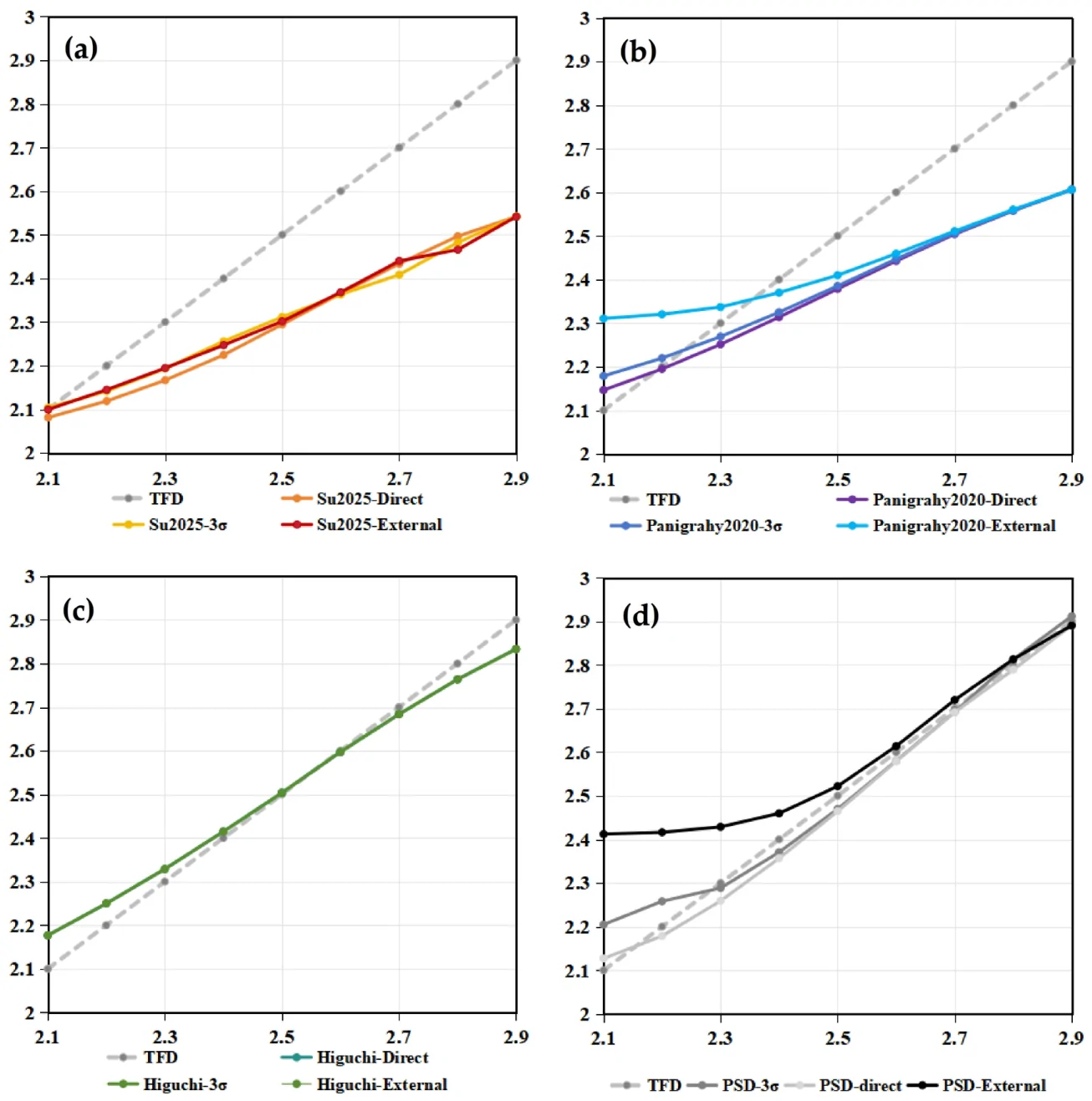
Estimated FD values of FBM images under different mapping strategies obtained by the four algorithms: (**a**) Su2025; (**b**) Panigrahy2020; (**c**) Higuchi; (**d**) PSD.

Table 1 summarizes the estimated FD values $D_{est}$ obtained by the four algorithms, namely Su2025-DBC, Panigrahy2020-DBC, Higuchi, and PSD, under direct mapping, 3*σ* statistical mapping, and external-coefficient mapping strategies. The corresponding regression parameters between $D_{est}$ and TFD are also reported, including the correlation coefficient, slope, and intercept. Figure 3 was plotted to visually observe the deviation between $D_{est}$ values and the TFD values.

Overall, the estimated FD values showed strong linear relationships with TFD. The correlation coefficients ranged from 0.9599 to 0.9995, indicating that all four algorithms were able to capture changes in FBM surface complexity. However, a high correlation does not necessarily imply accurate estimation. The regression slope and intercept provide a more direct indication of systematic bias. Ideally, $D_{est}=TFD$, corresponding to a slope close to 1 and an intercept close to 0.

The DBC-based algorithms exhibited clear deviations from the ideal slope and relatively large positive intercepts under all three linear mapping strategies. Under direct mapping, for example, the regression slopes of Su2025-DBC and Panigrahy2020-DBC were 0.6087 and 0.5931, respectively, while the intercepts were 0.7806 and 0.8943. These results indicate that both algorithms can track the overall increasing trend of TFD, but their sensitivity to changes in TFD is attenuated. As a result, some low-FD surfaces tend to be overestimated, whereas high-FD surfaces tend to be increasingly underestimated. When the preprocessing strategy changed from direct mapping to 3*σ* statistical mapping or external-coefficient mapping, the slopes of both DBC methods generally decreased, while their intercepts increased. This worsening bias suggests that changes in the linear mapping strategy distort the fractal dimension estimation.

The Higuchi method exhibited a remarkably consistent estimation pattern across the three linear mapping strategies. Its regression slope remained approximately 0.843, the intercept remained near 0.40, and the correlation coefficient was approximately 0.9995 in all cases. This reveals that the Higuchi method is largely insensitive to the different mapping strategies. However, such stability reflects robustness to linear mapping rather than absence of bias. The slope remained below 1 and the intercept above 0, indicating that the method still exhibited systematic FD bias for the mapped FBM images.

The PSD method was strongly affected by the linear mapping strategy. FD results under direct mapping produced the best regression performance, with a slope of 0.9955 and an intercept of −0.0070. Under the 3*σ* statistical mapping strategy, the slope decreased to 0.9180 and the intercept increased to 0.2149. Under the external-coefficient mapping strategy, the performance deteriorated further, with the slope decreasing to 0.6400 and the intercept increasing to 0.9862. The correlation coefficient also dropped from 0.9969 under direct mapping to 0.9599 under external-coefficient mapping. These results show that although the PSD method can provide accurate estimates under specific mapping conditions, its performance is sensitive to linear mapping strategies.

The influence of the linear mapping strategy on the PSD method is comparable to that on Su2025-DBC and Panigrahy2020-DBC. In all three cases, the best fractal dimension estimation accuracy is achieved under the direct mapping strategy, followed by the 3*σ* statistical mapping and external-coefficient mapping strategies. Furthermore, these results demonstrate that even for the same method, FBM images generated with different linear mapping strategies yield distinct computed FD results, confirming that the linear mapping strategy alters the grayscale properties of the input images.

**Figure 4:**
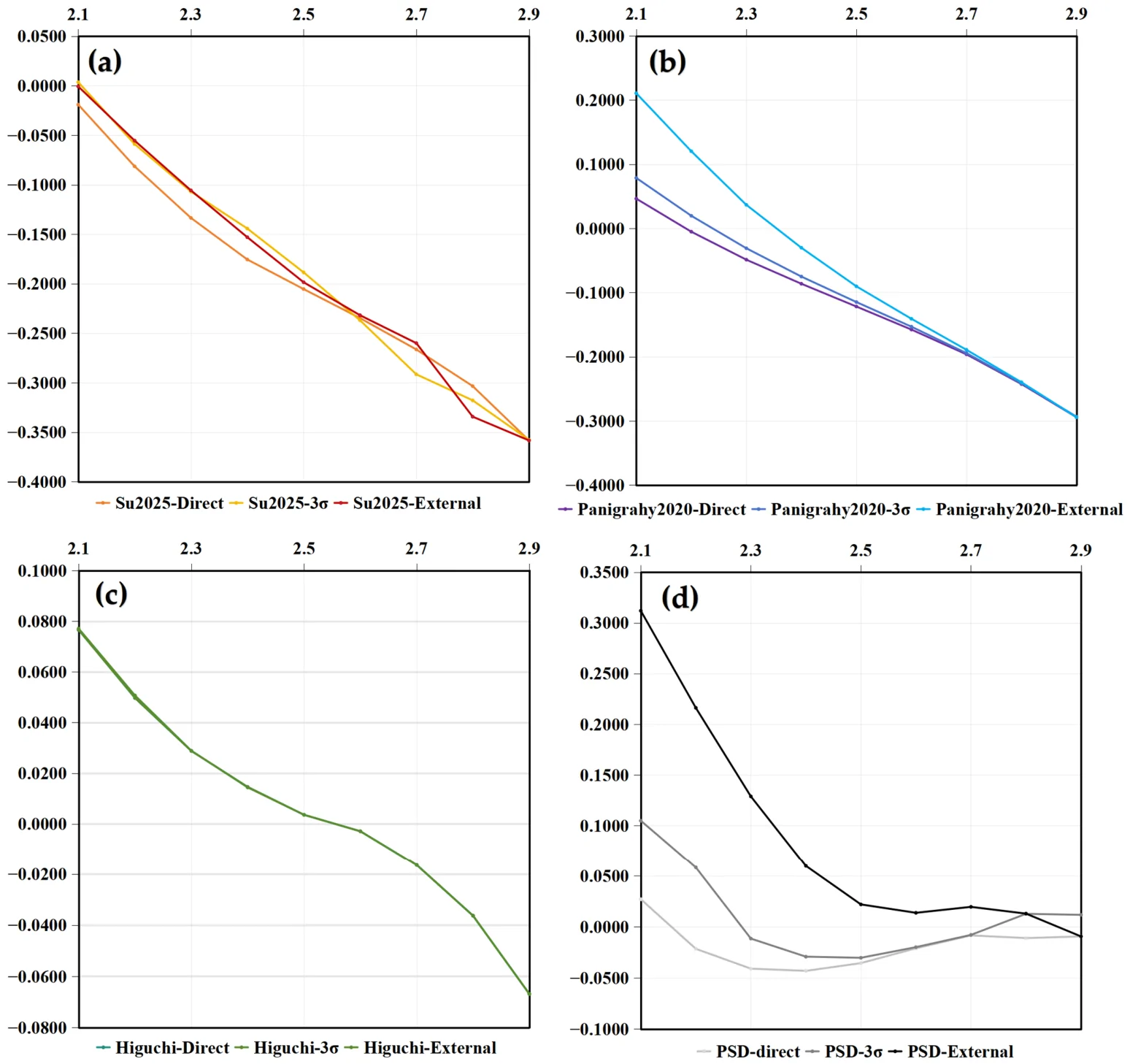
Signed errors (SE) of FD estimates from FBM images processed via three linear mapping strategies. (**a**) Su2025; (**b**) Panigrahy2020; (**c**) Higuchi; (**d**) PSD.

To further evaluate FD estimation accuracy, Table 2 reports the signed errors (SE) of $D_{est}$ with respect to TFD for the four algorithms under the three linear mapping strategies. Consistent with the regression results, the error distributions differed markedly among algorithms, and the optimal mapping strategy was not fixed. The relationships between signed errors and TFD values are presented in Figure 4.

For Su2025-DBC, the absolute SE increased monotonically with TFD. In the low-FD range (TFD=2.1–2.3), the external-coefficient strategy yielded the smallest errors. In the intermediate and high-FD ranges, the best-performing strategy shifted among 3*σ* statistical mapping, direct mapping, and external-coefficient mapping. These results suggest that under the current experimental conditions, Su2025-DBC is more suitable for lower-complexity FBM surfaces. For surfaces with higher fractal dimensions, its box-counting process may not respond sufficiently to increasing surface roughness.

The Panigrahy2020-DBC method showed a similar increase in SE with TFD, but the choice of the optimal linear mapping strategy was more complex. At TFD = 2.1–2.2, direct mapping gave the lowest errors. At TFD = 2.3 & 2.9, 3*σ* statistical mapping performed best. Over the broader range of TFD = 2.4–2.8, external-coefficient mapping consistently produced the lowest errors. This pattern indicates that the error of improved DBC methods is determined not only by the linear mapping strategy, but also by images’ TFD values. Although Table 1 reveals that images generated by the direct mapping strategy performed best in FD estimation overall, this performance also varies with the TFD of the images.

The Higuchi method showed error behavior consistent with its regression stability. The error curves under the three linear mapping strategies almost overlapped. The largest errors occurred at *TFD* = 2.1–2.2. The error then decreased rapidly and reached its lowest level in the range *TFD* = 2.4–2.7. At *TFD* = 2.8–2.9, the error increased again. Therefore, the main advantage of the Higuchi method lies in its robustness to preprocessing, rather than consistently achieving the lowest error across all FD levels.

The PSD method exhibited strong strategy dependence and clear variation across FD ranges. In the low-FD range (*TFD* = 2.1–2.2), direct mapping produced the lowest errors, whereas external-coefficient mapping led to much larger errors. As TFD increased, the errors under the three linear mapping strategies gradually converged and became relatively small in the high-FD range. The best mapping strategy also changed with TFD: 3*σ* statistical mapping strategy performed best at *TFD* = 2.3–2.4, external-coefficient mapping performed best at *TFD* = 2.5–2.6, and all strategies yielded absolute errors below 0.02 when *TFD* > 2.6. This suggests that the PSD method is more sensitive to grayscale changes for low-complexity surfaces, while the power-law spectral structure becomes more stable as surface TFD increases.

The preceding analysis reveals that no single linear mapping strategy is universally optimal across all algorithms and TFD values. Although the error data in Table 2 support the discussion of the optimal strategy in terms of accuracy relative to TFD, considering the inherent errors of fractal dimension algorithms, what appears to be optimal may instead reflect a compensation between algorithmic bias and mapping-induced error, rather than actual superiority of the mapping strategy itself. Therefore, the effect of linear mapping strategies should be evaluated based on the error variation across different mapping strategies.

Figure 4 further shows that while linear mapping alters the estimated fractal dimensions, the overall trends remain largely consistent across strategies for each FD algorithm. Using the FD results of direct mapping as a baseline, for Su2025-DBC, the error curves of the other two strategies shift upward at low fractal dimensions and begin to slope downward as TFD increases. For Panigrahy2020-DBC, a similar upward deviation is observed at low TFD values, but its magnitude gradually diminishes as TFD increases. The Higuchi method remains remarkably stable, exhibiting negligible differences among the three strategies. The PSD method exhibits a pattern comparable to that of Panigrahy2020-DBC: an upward shift at low TFD values, followed by a gradual convergence as TFD increases. Overall, for each method, as TFD approaches 2.9, the errors of the three strategies converge, whereas larger deviations are predominantly concentrated in the low-fractal-dimension region. These results indicate that, for a given fractal dimension algorithm, the choice of linear mapping strategy represents a systematic source of variation in the estimated fractal dimension values.

### 3.2. The Goodness-of-Fit Analysis Within the FD Algorithms

Section 3.1 demonstrated that both FD estimates and their associated errors varied across linear mapping strategies. The present analysis examines whether these differences can be explained by the internal goodness-of-fit of each algorithm. The internal $R^{2}$ quantifies the linearity of the algorithm-specific log-log regression, and therefore reflects the consistency of the scaling law used for FD estimation. However, it should not be interpreted as a direct measure of estimation accuracy. A highly linear log-log relationship may still yield a biased FD estimate if its slope is systematically affected by preprocessing. Table 3 reports the internal goodness-of-fit $R^{2}$ values obtained during FD estimation, along with correlation coefficients (between internal goodness-of-fit and FD absolute SE).

As shown in Table 3, Su2025-DBC produced the highest $R^{2}$ values under direct mapping, generally exceeding 0.99923. Considering the low correlation coefficient of 0.6165, the FD estimation errors of Su2025-DBC may be influenced not solely by goodness of fit. More importantly, the high $R^{2}$ under direct mapping did not eliminate slope compression in the $D_{est}-TFD$ regression or the increased error at high TFD values. This suggests that the primary limitation is unlikely to be a failure of log-log fitting.

Panigrahy2020-DBC exhibited similar phenomena but with a different trend. Its internal goodness of fit was relatively lower under direct mapping and higher under the 3*σ* statistical mapping strategy and external-coefficient mapping. Nevertheless, when the fitting results are examined alongside the FD estimation errors, a high $R^{2}$ does not necessarily correspond to a lower estimation error (see Table 1 and Table 3). The correlation coefficient (Table 3) was negative, with an absolute value exceeding 0.8, indicating a relatively strong negative correlation between goodness of fit and absolute error. Considering that the goodness-of-fit values were all greater than 0.9991 and the relative FD error was as high as 10% (calculated from Table 2), the influence of goodness of fit remains limited. This discrepancy indicates that improving the linearity of the internal box-counting regression does not necessarily improve agreement with the theoretical FD.

The Higuchi method showed exceptionally stable goodness of fit across all preprocessing strategies, with $R^{2}$ > 0.99989. Differences among the three linear mapping strategies were negligible. This behavior agrees with the nearly overlapping regression parameters and FD estimation errors observed in Section 3.1. However, the stability of $R^{2}$ again indicates robustness to preprocessing rather than complete absence of estimation bias. The correlation coefficient in Table 3 shows that there is no linear correlation between goodness of fit and absolute FD error for the Higuchi method.

The PSD method yielded lower internal $R^{2}$ values than the other three methods. As TFD varied, the best goodness of fit alternated among the three mapping strategies. However, considering the correlation coefficient in Table 3, the FD estimation errors may be influenced not solely by goodness of fit but also by other mapping-induced changes.

Taken together, these results show that the optimal gray-level mapping strategy depends on both the FD algorithm and the target FD range. Notably, high fitting accuracy may sometimes arise from compensating errors rather than genuinely accurate FD estimation. Overall, the internal *R*^2^ analysis shows that goodness of fit is a necessary but insufficient indicator of FD estimation reliability. A high internal *R*^2^ confirms that an algorithm yields a stable scaling relationship, but it does not guarantee that the resulting slope is unbiased with respect to TFD.

### 3.3. Effects of Linear Mapping on Image Statistical Properties

The preceding sections established that FD estimates, errors, and internal goodness of fit vary jointly with the mapping strategy and the estimation algorithm (Section 3.1 and Section 3.2). As each algorithm in this study receives an 8-bit grayscale image as input, systematic differences in the image’s statistical properties across mapping strategies are therefore a candidate source for FD estimation errors. This section characterizes how the three mapping strategies transform the basic statistical properties of FBM images.

Table 4 reports the mean, standard deviation $\sigma_{Z}$, and dynamic range ($Z_{min}$ and ${\;Z}_{max}$) of the original FBM matrices and the corresponding mapped images across all TFD levels (2.1–2.9) and mapping strategies. FBM surfaces with different theoretical fractal dimensions thus differ not only in spatial roughness but also in their numerical amplitude distributions. Linear mapping transforms this original statistical structure into the 8-bit grayscale distributions that serve as input to the FD estimation algorithms examined in this study.

The direct mapping strategy maps the minimum and maximum pixel values of each image to 0 and 255, respectively. As a result, all images occupy the full 8-bit grayscale dynamic range. This strategy maximizes single-image contrast, but it also normalizes away the original amplitude-scale differences among samples. The results show that, under direct mapping, the image mean decreased from 155.07 to 121.08 and the standard deviation decreased from 50.01 to 29.45 as TFD increased from 2.1 to 2.9 (Table 4). Nevertheless, the direct method exhibits significantly larger dynamic range and standard deviation across TFD values than the other two strategies.

3*σ* statistical mapping determines the mapping coefficients from the mean and standard deviation. After mapping, the image mean remained close to 127.50 and the standard deviation close to 26.50 across all TFD levels. This strategy reduces the influence of extreme values on the mapping coefficients and stabilizes the dominant gray-level distribution. At the same time, the output dynamic range still increased substantially with TFD, from approximately 45–180 to 19–248. This indicates that the proportion and spread of extreme gray-level values change as the surface fractal dimension increases, even though the central distribution is stabilized.

External-coefficient mapping uses a single set of mapping coefficients for the entire batch. It therefore preserves the relative amplitude differences among samples. Under this strategy, the image mean, standard deviation, and dynamic range all changed with TFD, more closely following the statistical evolution of the original FBM matrices. However, for low-FD samples, the original dynamic range was small, and the mapped images occupied only a narrow grayscale interval. This represents the most severe dynamic-range compression among the three strategies.

The three strategies thus produce images with fundamentally different statistical structures from the same set of FBM matrices—differences in dynamic range, mean intensity, and grayscale dispersion that persist across the full TFD range (Table 4). Each strategy embodies a distinct priority: single-image contrast maximization (direct), central-distribution stabilization (3*σ*), or inter-sample amplitude conservation (external-coefficient). Because FD estimation algorithms differ in their sensitivity to the grayscale range and dispersion properties of their input, the mapping strategy is expected to produce algorithm-dependent effects on FD estimates.

### 3.4. Linear Mapping Fidelity and Quantization Error

Section 3.3 demonstrated that the three mapping strategies produce substantially different grayscale statistical profiles. An alternative explanation for the observed differences in FD estimates, however, is that the linear mapping and integer quantization steps themselves introduce numerical distortion sufficient to bias downstream computation. This section tests that hypothesis by evaluating mapping fidelity through three complementary measures: linear correlation preservation, residual statistics, and relative quantization burden.

As shown in Table 5, all three strategies preserved a strong linear relationship between the original FBM matrices and the mapped grayscale images. The Pearson correlation coefficients were consistently higher than 0.99908. Direct mapping produced the highest correlations, although they decreased slightly as TFD increased. The 3*σ* statistical mapping showed slightly lower but nearly constant correlations. The external-coefficient mapping gave the lowest correlation at low TFD, but the value increased with TFD. The spatial structure of the original FBM surfaces was essentially preserved with negligible linear distortion under all conditions.

The residual, defined as the difference between the theoretical mapped value (*a***X** + *b*) and the actual 8-bit quantized value, quantifies the numerical error introduced by the mapping and quantization operations. The residual mean reflects potential systematic bias introduced by linear mapping and integer quantization. As shown in Table 5, the absolute residual mean was below 0.001540 for images under all linear mapping strategies and all TFD values. Relative to the 8-bit grayscale range of 0–255, this bias is negligible. The residual standard deviation measures the dispersion of quantization error. For all three strategies, it remained within a narrow range of 0.2882–0.2894, close to the theoretical level expected for uniform rounding error $\left(\sqrt{1/12}\approx 0.2887\right)$. Both metrics indicate that the absolute errors introduced by linear mapping are relatively limited.

However, the absolute residual magnitude alone is insufficient to describe the effective perturbation introduced by quantization. The same absolute residual standard deviation of approximately 0.289 gray levels represents markedly different perturbations depending on the statistical scale of the mapped image. As shown in Table 4, the three strategies produced markedly different dynamic ranges and standard deviations. The same residual standard deviation may therefore correspond to different relative error levels. To account for this effect, the quantization error can be interpreted relative to the image standard deviation. Therefore, the relative residual $\eta_{\sigma }$ was introduced:

(10) $$\eta_{\sigma }=\frac{\sigma_{e}}{\sigma_{Z}}$$

where $\sigma_{e}$ is the residual standard deviation from Table 5 and $\sigma_{Z}$ is the standard deviation of the mapped image from Table 4. $F_{1}$ and $F_{2}$ are the ratios of the relative residuals from the 3*σ* statistical strategy and external-coefficient strategy to the relative residuals from direct mapping strategy, respectively. The results of $\eta_{\sigma }$ and amplification factors ($F_{1}$ & $F_{2}$) are listed in Table 6.

As shown in Table 6, all three linear mapping strategies introduce relative residuals, whose values vary with both the strategy and the fractal dimension. Under the direct mapping strategy, the proportion of introduced relative residuals increases gradually; under the 3*σ* strategy, it remains stable; and under the external-coefficient strategy, it shows a decreasing trend. Compared to direct mapping, both the 3*σ* and external-coefficient strategies introduce larger relative residuals, with the external strategy producing significantly larger residuals than the 3*σ* strategy (except at *TFD* = 2.9), reaching up to 7.42 times (External, *TFD* = 2.1). Moreover, as the theoretical fractal dimension increases, the proportions of introduced residuals across the three strategies gradually converge. Thus, all three strategies introduce errors relative to the original image matrix, but the differing relative residuals cause the characteristics of the fractal dimension image—on which the calculations are based—to change with the linear mapping strategy.

To further assess how the variation of relative residuals relates to fractal dimension differences across strategies, two steps were taken. First, the fractal dimension changes for the 3*σ* and external strategies relative to direct mapping were calculated. Second, the correlation between these relative FD changes and the amplification factors $F_{1}$ and $F_{2}$ (Table 6) was examined. This analysis scheme isolated the fractal dimension variation caused solely by differences in linear mapping strategies. Results were listed in Table 7.

Table 7 shows that both the 3*σ* and external strategies yield fractal dimension values that deviate from those of the direct strategy, but the pattern of deviation varies across the fractal dimension algorithms.

For Su2025-DBC, the correlation coefficient between the relative FD deviations of both strategies and the amplification factors ($F_{1}{\;\&\;F}_{2}$) exceeds 0.87, indicating a strong positive correlation. This means that, for FBM images with different TFD values, the larger the relative residuals introduced by the linear mapping, the greater the positive deviation in the fractal dimension. When both $F_{1}$ and $F_{2}$ gradually decrease with increasing TFD values, the impact of relative residuals diminishes correspondingly. Under the 3*σ* strategy, the positive deviation gradually declines between TFD 2.1 and 2.5; under the external-coefficient strategy, this decline occurs between TFD 2.1 and 2.6. For TFD beyond 2.6, the drop in relative residuals allows other factors to begin exerting influence, such as deviations in standard deviation and dynamic range. A larger relative residual contributes more significantly to the deviation of the fractal dimension value. The external strategy, which yields higher relative residuals than the 3*σ* strategy, also exhibits a higher correlation coefficient of relative FD variation and the amplification factors ($F_{2}$), which indicates that more FD deviations are caused by the introduced relative residuals.

For Panigrahy2020-DBC, the correlation coefficients between fractal dimension deviation and relative residual amplification exceed 0.92, indicating a significant positive relationship: a larger introduced residual leads to a larger fractal dimension deviation. As TFD increases, the introduced relative residual gradually decreases, and the relative FD variation under both strategies monotonically decreases accordingly. In Table 7, the FD deviations under the external-coefficient and 3*σ* statistical strategies for the Panigrahy2020-DBC method show a strict correlation with the trend of relative residual in Table 6. As noted in Section 2.3, although Panigrahy2020 is also a DBC-based method, it employs multiple optimization strategies to reduce biases from box undercounting and log-log fitting, and the results suggest that Panigrahy2020 better reflects the intrinsic characteristics of the mapped images.

For the Higuchi method, the FD deviation shows no clear correlation with the amplification factors. Under the 3*σ* strategy, the fractal dimension remains almost unchanged, with only a small deviation at *TFD* = 2.1, where the residual amplification factor is largest. Under the external strategy, a slightly larger deviation occurs only at *TFD* = 2.1–2.4. This suggests that the Higuchi method is insensitive to most of the introduced relative residuals. As discussed in Section 2.3, unlike DBC-based methods, Higuchi involves extensive summation and averaging in the calculation of A(k). Since the absolute noise values are zero-mean, random, and limited in fluctuation range, rounding errors are gradually averaged away. Consequently, the introduced residuals do not produce as significant FD deviations as in the other algorithms.

For the PSD method, the correlation coefficients between FD deviation and amplification factors reach 0.97 under the external-coefficient strategy but drop to approximately 0.72 under the 3*σ* statistical strategy. Examining the specific trends, under the external-coefficient strategy, the FD deviation decreases monotonically with the amplification factor as TFD increases. Under the 3*σ* strategy, it decreases monotonically from TFD 2.1 to 2.7 but rises again at TFD 2.8 and 2.9. This likely arises from a similar cause as in the previous Su2025 methods: under the external strategy, where relative residuals are larger, residuals dominate the FD variation, yielding a high correlation coefficient. Under the 3*σ* statistical strategy, the smaller relative residuals lead to a reduced influence on FD deviation.

Additionally, at TFD 2.9, according to Table 4 and Table 5, the image matrices produced by the three linear mapping strategies are very similar in terms of dynamic range, standard deviation, and relative residual. Correspondingly, the FD deviations across linear mapping strategies are minimal.

Taken together, the three linear mapping strategies all introduce residuals into the image matrix, primarily caused by rounding errors. The introduced relative residual serves as a metric to evaluate these errors. Differences in linear mapping strategies lead to variations in image standard deviation, which in turn result in different relative residuals. By comparing the relative residual differences with the corresponding FD differences, we find that, for a given fractal dimension algorithm, the linear mapping strategy alters the relative residual, thereby affecting the fractal dimension results. For the two DBC-based methods and the PSD method, the relative residual leads to an increase in fractal dimension, and these two variables show a positive correlation. When the relative residual is small, its effect on fractal dimension variation is correspondingly reduced. Furthermore, different fractal dimension algorithms exhibit varying sensitivity to the relative residual: the Higuchi method is the most robust, while the PSD method is the most affected, with a maximum relative FD deviation of 13.4%.

## 4. Conclusions

Linear gray-level mapping for 8-bit conversion of continuous FBM has commonly been considered an innocuous formatting step in FD validation, but its systematic influence remained uncharacterized. This work assessed that influence across three linear mapping strategies and four FD algorithms on identical synthetic FBM surfaces.

The mapping strategy produced algorithm- and TFD-dependent variations in FD estimates. The PSD method was the most affected, with its regression slope against TFD decreasing from 0.9955 (direct) to 0.6400 (external-coefficient). The two DBC variants displayed compressed slopes and increased intercepts under the 3*σ* statistical and external-coefficient strategies relative to direct mapping. The Higuchi method was essentially insensitive to mapping strategy.

These shifts did not result from degraded scaling behavior. Internal goodness of fit exceeded 0.998 for the DBC variants and Higuchi, and 0.982 for PSD, with no systematic relationship to FD error, ruling out fitting quality as the source of the observed bias.

Instead, the three strategies produced images with substantially different grayscale statistics (standard deviation and dynamic range) from the same FBM surface. The absolute quantization error was small (*σ* ≈ 0.289), but the resulting relative perturbation (quantified as the relative residual) differed by up to 7.42-fold across strategies, driven by differences in image standard deviation.

The relative residual proved effective as a cross-algorithm metric. FD deviations from the direct mapping baseline correlated strongly with relative residual amplification for both DBC variants (*r* > 0.87) and PSD (*r* up to 0.97), confirming it as the primary pathway through which mapping propagates into estimation error. The Higuchi method showed limited correlation, reflecting its intrinsic insensitivity to grayscale dispersion. This correlation was not uniform across the full range of relative residual values: when the relative residual was small (e.g., under the 3*σ* statistical mapping strategy), the association weakened. This dose-dependent relationship implies that keeping the relative residual below an algorithm-specific threshold ensures FD comparisons primarily reflect algorithmic performance. The exact threshold is algorithm-dependent; PSD was highly sensitive, whereas Higuchi remained robust across the tested range.

These findings establish that linear gray-level mapping violates the assumption of benchmark neutrality: it constitutes a confounding variable, not a neutral preprocessing step. Omitting mapping parameters from validation studies, therefore, compromises the comparability of published benchmark results.

We recommend that FD benchmarking studies explicitly report the mapping strategy and its associated relative residual. Minimizing the relative residual by choosing strategies that preserve image standard deviation distinguishes algorithmic performance from preprocessing effects in fractal dimension evaluations.

It should be noted that the conclusions of this study are derived from synthetic FBM images and are applicable to the study of FD algorithms that use FBM images as a benchmark. Real-world images, however, typically do not satisfy the strict self-affinity and Gaussian grayscale statistics that characterize FBM. The influence of linear mapping strategies on FD estimation for such images therefore requires further investigation.

## Figures and Tables

**Table 1:** FD estimates of FBM images under different mapping strategies, obtained by the four algorithms, and the corresponding regression parameters (correlation coefficient, slope, and intercept) (The optimal results are highlighted in bold).

	Su2025			Panigrahy2020			Higuchi			PSD		
TFD	Direct	3*σ*	External Coefficient	Direct	3*σ*	External Coefficient	Direct	3*σ*	External Coefficient	Direct	3*σ*	External Coefficient
2.1	2.0809	2.1036	2.0993	2.1464	2.1787	2.3107	2.1767	2.1765	2.1771	2.1270	2.2048	2.4120
2.2	2.1186	2.1411	2.1446	2.1949	2.2197	2.3203	2.2497	2.2497	2.2507	2.1785	2.2582	2.4162
2.3	2.1665	2.1931	2.1943	2.2513	2.2691	2.3368	2.3288	2.3288	2.3289	2.2591	2.2885	2.4288
2.4	2.2245	2.2558	2.2470	2.3138	2.3250	2.3699	2.4146	2.4146	2.4144	2.3569	2.3708	2.4597
2.5	2.2946	2.3115	2.3015	2.3783	2.3853	2.4098	2.5036	2.5036	2.5036	2.4645	2.4697	2.5219
2.6	2.3653	2.3631	2.3682	2.4424	2.4469	2.4592	2.5971	2.5971	2.5971	2.5790	2.5801	2.6137
2.7	2.4336	2.4085	2.4400	2.5038	2.5057	2.5109	2.6836	2.6836	2.6836	2.6916	2.6921	2.7195
2.8	2.4966	2.4822	2.4657	2.5575	2.5585	2.5603	2.7637	2.7637	2.7637	2.7890	2.8127	2.8129
2.9	2.5417	2.5423	2.5417	2.6058	2.6067	2.6058	2.8331	2.8331	2.8331	2.8906	2.9117	2.8906
Correlation	0.9971	**0.9984**	0.9978	**0.9993**	0.9990	0.9832	0.9995	0.9995	0.9995	**0.9969**	0.9882	0.9599
Slope	**0.6087**	0.5527	0.5576	**0.5931**	0.5539	0.3897	0.8433	**0.8434**	0.8425	**0.9955**	0.9180	0.6400
Intercept	**0.7806**	0.9295	0.9175	**0.8943**	1.0036	1.4573	0.3974	**0.3972**	0.3996	**−0.0070**	0.2149	0.9862

**Table 2:** Signed errors (SEs) of FD estimates from FBM images processed via three linear mapping strategies (The optimal results are highlighted in bold).

	Su2025			Panigrahy2020			Higuchi			PSD		
TFD	Direct	3*σ*	External Coefficient	Direct	3*σ*	External Coefficient	Direct	3*σ*	External Coefficient	Direct	3*σ*	External Coefficient
2.1	−0.0191	0.0036	**−0.0007**	**0.0464**	0.0787	0.2107	0.0767	**0.0765**	0.0771	**0.0270**	0.1048	0.3120
2.2	−0.0814	−0.0589	**−0.0554**	**−0.0051**	0.0197	0.1203	**0.0497**	**0.0497**	0.0507	**−0.0215**	0.0582	0.2162
2.3	−0.1335	−0.1069	**−0.1057**	−0.0487	**−0.0309**	0.0368	**0.0288**	**0.0288**	0.0289	−0.0409	**−0.0115**	0.1288
2.4	−0.1755	**−0.1442**	−0.1530	−0.0862	−0.0750	**−0.0301**	0.0146	0.0146	**0.0144**	−0.0431	**−0.0292**	0.0597
2.5	−0.2054	**−0.1885**	−0.1985	−0.1217	−0.1147	**−0.0902**	0.0036	0.0036	0.0036	−0.0355	−0.0303	**0.0219**
2.6	−0.2347	−0.2369	**−0.2318**	−0.1576	−0.1531	**−0.1408**	−0.0029	−0.0029	−0.0029	−0.0210	−0.0199	**0.0137**
2.7	−0.2664	−0.2915	**−0.2600**	−0.1962	−0.1943	**−0.1891**	−0.0164	−0.0164	−0.0164	−0.0084	**−0.0079**	0.0195
2.8	**−0.3034**	−0.3178	−0.3343	−0.2425	−0.2415	**−0.2397**	−0.0363	−0.0363	−0.0363	**−0.0110**	0.0127	0.0129
2.9	−0.3583	**−0.3577**	−0.3583	−0.2942	**−0.2933**	−0.2942	−0.0669	−0.0669	−0.0669	**−0.0094**	0.0117	**−0.0094**

**Table 3:** Internal goodness-of-fit values and the corresponding PE of the four FD algorithms (PE: Pearson correlation coefficient between internal goodness-of-fit and FD absolute errors; the optimal results are highlighted in bold).

	Su2025			Panigrahy2020			Higuchi			PSD		
TFD	Direct	3*σ*	External Coefficient	Direct	3*σ*	External Coefficient	Direct	3*σ*	External Coefficient	Direct	3*σ*	External Coefficient
2.1	**0.99925**	0.99909	0.99914	0.99982	**0.99999**	0.99951	0.99998	0.99998	0.99998	**0.99203**	0.98668	0.98269
2.2	**0.99923**	**0.99923**	0.99897	0.99972	**0.99991**	0.99986	0.99998	0.99998	0.99998	0.99353	**0.99504**	0.98434
2.3	**0.99938**	0.99937	0.99904	0.99961	0.99979	**0.99999**	0.99998	0.99998	0.99998	**0.99407**	0.99301	0.98844
2.4	**0.99949**	0.99921	0.99896	0.99950	0.99964	**0.99993**	0.99997	0.99997	0.99997	0.99385	0.99353	**0.99427**
2.5	**0.99953**	0.99894	0.99887	0.99939	0.99949	**0.99974**	0.99996	0.99996	0.99996	0.99284	0.99276	**0.99594**
2.6	**0.99956**	0.99928	0.99914	0.99929	0.99935	**0.99951**	0.99994	0.99994	0.99994	0.99058	0.99056	**0.99417**
2.7	**0.99955**	0.99941	0.99944	0.99920	0.99924	**0.99931**	0.99993	0.99993	0.99993	0.98827	0.98828	**0.99157**
2.8	**0.99952**	0.99944	0.99942	0.99916	0.99918	**0.99920**	0.99990	0.99990	0.99990	0.98874	**0.99261**	0.99260
2.9	**0.99957**	0.99949	**0.99957**	0.99913	**0.99914**	0.99913	0.99989	0.99989	0.99989	0.98736	**0.99161**	0.98736
PE		−0.6165			−0.8820			−0.0709			−0.6165	

**Table 4:** Mean, standard deviation and dynamic ranges of the original FBM matrices and images processed by the three linear mapping strategies (The original values × 10^−7^).

	Mean				Standard Deviation $\sigma_{Z}$				Minimum $Z_{min}$				Maximum $Z_{max}$			
TFD	Original	Direct	3*σ*	External	Original	Direct	3*σ*	External	Original	Direct	3*σ*	External	Original	Direct	3*σ*	External
2.1	3.56	155.0695	127.5004	119.0524	2.34	50.0064	26.5013	6.7495	−3.69	0	45	98	8.23	255	180	133
2.2	3.63	151.2486	127.5003	119.2631	2.61	48.2799	26.5013	7.5470	−4.55	0	44	96	9.25	255	184	135
2.3	3.71	147.6261	127.5003	119.4878	2.96	45.8479	26.5025	8.5447	−5.82	0	42	92	10.64	255	190	139
2.4	3.79	143.8016	127.5008	119.7270	3.40	43.2211	26.5018	9.8188	−7.52	0	39	87	12.54	255	196	145
2.5	3.88	138.9421	127.5004	119.9760	3.98	40.1580	26.5004	11.4877	−9.89	0	36	80	15.38	255	204	153
2.6	3.97	134.0451	127.4995	120.2403	4.76	37.0318	26.5017	13.7490	−13.27	0	32	70	19.53	255	214	165
2.7	4.07	129.2996	127.4992	120.5135	5.87	34.0322	26.5016	16.9570	−18.26	0	27	56	25.77	255	225	183
2.8	4.16	124.9189	127.5005	120.7955	7.54	31.4135	26.5016	21.7722	−25.83	0	22	34	35.40	255	237	211
2.9	4.26	121.0789	127.5006	121.0789	10.20	29.4459	26.5010	29.4459	−37.69	0	19	0	50.67	255	248	255

**Table 5:** Pearson correlation coefficients and residual statistics between the original matrices and images processed by the three linear mapping strategies (The optimal results are highlighted in bold).

TFD	Pearson Correlation Coefficient			Residual Mean			Residual Standard Deviation		
	Direct	3*σ*	External	Direct	3*σ*	External	Direct	3*σ*	External
2.1	**0.999983**	0.999941	0.999081	**0.000006**	0.000362	−0.000338	0.2889	**0.2886**	0.2894
2.2	**0.999982**	0.999941	0.999264	0.000514	**0.000305**	−0.000538	0.2885	**0.2884**	0.2894
2.3	**0.999980**	0.999941	0.999428	**0.000063**	0.000259	−0.000196	**0.2886**	0.2888	0.2890
2.4	**0.999978**	0.999941	0.999567	**0.000375**	0.000805	0.001195	**0.2882**	0.2888	0.2888
2.5	**0.999974**	0.999941	0.999684	**−0.000044**	0.000427	−0.000884	0.2892	**0.2887**	0.2889
2.6	**0.999970**	0.999941	0.999780	**0.000116**	−0.000546	0.000148	0.2886	0.2885	**0.2884**
2.7	**0.999964**	0.999941	0.999855	−0.001210	−0.000820	**−0.000436**	0.2890	**0.2885**	0.2890
2.8	**0.999958**	0.999941	0.999912	0.000642	0.000469	**0.000024**	0.2889	**0.2882**	0.2883
2.9	**0.999952**	0.999940	**0.999952**	−0.001539	**0.000645**	−0.001539	**0.2887**	0.2892	**0.2887**

**Table 6:** The relative residuals and amplification factors $F_{1}$ and $F_{2}$ vary along the TFD values (The optimal results are highlighted in bold).

TFD	$\eta_{\sigma }=\sigma_{e}/\sigma_{Z}$				
	Direct	3*σ*	$F_{1}$ = 3*σ*/Direct	External	$F_{2}$ = External/Direct
2.1	**0.58%**	1.09%	1.88	4.29%	7.42
2.2	**0.60%**	1.09%	1.82	3.83%	6.42
2.3	**0.63%**	1.09%	1.73	3.38%	5.37
2.4	**0.67%**	1.09%	1.63	2.94%	4.41
2.5	**0.72%**	1.09%	1.51	2.51%	3.49
2.6	**0.78%**	1.09%	1.40	2.10%	2.69
2.7	**0.85%**	1.09%	1.28	1.70%	2.01
2.8	**0.92%**	1.09%	1.18	1.32%	1.44
2.9	**0.98%**	1.09%	1.11	0.98%	1.00

**Table 7:** Relative FD errors of the 3*σ* statistical and external-coefficient linear mapping strategies relative to direct mapping, and the Pearson correlation coefficients (PF) between these FD errors and the amplification factors ($F_{1}$ and $F_{2}$) (The optimal results are highlighted in bold).

	Su2025		Panigrahy2020		Higuchi		PSD	
TFD	3*σ* vs. Direct	External vs. Direct	3*σ* vs. Direct	External vs. Direct	3*σ* vs. Direct	External vs. Direct	3*σ* vs. Direct	External vs. Direct
2.1	2.27%	1.84%	1.50%	7.65%	−0.01%	0.02%	3.66%	13.40%
2.2	1.06%	1.23%	1.13%	5.71%	0.00%	0.04%	3.66%	10.91%
2.3	1.23%	1.28%	0.79%	3.80%	0.00%	0.00%	1.30%	7.51%
2.4	1.41%	1.01%	0.48%	2.42%	0.00%	−0.01%	0.59%	4.36%
2.5	0.74%	0.30%	0.29%	1.32%	0.00%	0.00%	0.21%	2.33%
2.6	−0.09%	0.12%	0.18%	0.69%	0.00%	0.00%	0.04%	1.35%
2.7	−1.03%	0.26%	0.08%	0.28%	0.00%	0.00%	0.02%	1.04%
2.8	−0.58%	−1.24%	0.04%	0.11%	0.00%	0.00%	0.85%	0.86%
2.9	0.02%	0.00%	0.03%	0.00%	0.00%	0.00%	0.73%	0.00%
PF	0.8740	**0.8900**	0.9270	**0.9711**	−0.5052	**0.6427**	0.7225	**0.9700**

## Data Availability

The original contributions presented in this study are included in the article. Further inquiries can be directed to the corresponding authors.

## Abbreviations

The following abbreviations are used in this manuscript:

DBC	Differential Box-Counting
FBM	Fractional Brownian Motion
FD	Fractal Dimension
GDI	Grid size is a divisor of image size
PE	Pearson correlation coefficient between goodness of fit and FD absolute error
PF	Pearson correlation coefficient between relative FD deviation and amplification factors
PSD	Power Spectral Density
SE	Signed Error; *SE* = $D_{est}$ − *TFD*
TFD	Theoretical Fractal Dimension; *TFD* = 3 − H
